# Creating behavioral engagement among higher education’s prospective students through social media marketing activities: The role of brand equity as mediator

**DOI:** 10.3389/fpsyg.2022.1004573

**Published:** 2022-10-06

**Authors:** Athapol Ruangkanjanases, Ornlatcha Sivarak, Ardy Wibowo, Shih-Chih Chen

**Affiliations:** ^1^Chulalongkorn Business School, Chulalongkorn University, Bangkok, Thailand; ^2^Mahidol University International College, Mahidol University, Nakhon Pathom, Thailand; ^3^College of Management, National Kaohsiung University of Science and Technology, Kaohsiung, Taiwan; ^4^Faculty of Economics and Business, Alma Ata University, Yogyakarta, Indonesia

**Keywords:** social media marketing activities, brand equity, behavioral engagement, higher education, digital natives

## Abstract

In today’s competitive environment, higher education needs to find an effective way to convey its brand to prospective students. Given that the “digital native” (Gen Z) is becoming college aged, social media marketing has become an essential approach to engage with them. However, blasting out recruiting content on its social feeds just isn’t working. By developing the higher education adjusted SMMA, structural equation modeling was adopted to figure out its effects on higher education brand equity and prospective student behavioral engagement, quantitatively. 356 3rd grade high school students in Indonesia were employed to assess the structural model. According to the findings of this study, SMMA has a considerable influence on brand equity and behavioral engagement, and brand equity has a noteworthy effect on behavioral engagement. Moreover, brand equity was found as a statistically meaningful mediator in the relationship between SMMA and behavioral engagement. The outcome advised the higher education need to organize its social in fun and interactive ways by leveraging higher education’s SMMA as a pillar or benchmark on arranging social media posts and content. Yet, the content and posts should still need prioritize institution awareness and the good image of a higher education. The theoretical and managerial implication were discussed further.

## Introduction

In today’s competitive market, higher education must become more market-oriented by actively communicating its brand to their prospective students (Sujchaphong et al., 2017). Social media, such as YouTube, Facebook, Instagram, Twitter, Tik Tok, blogs, and so on, is a favorable way to reach out to customers and get their attention (Li et al., 2020). Since the prospective customers of higher education are generation Z—who were born from 1997 to 2012 and are commonly referred to as “digital natives” and heavily influenced by early access to the internet and online communication—connecting and engaging them through social media is promising (Seemiller and Grace, 2016). Even more, they shift their information-seeking behavior by using social media to seek particular information (Hamid et al., 2016).

Many factors play a role on prospective higher education students’ decision about which college they want to attend. Factors such as institution recognition, learning environment, and country image (Hailat et al., 2021), educational establishments (James-MacEachern and Yun, 2017), employability, and community outreach (Miotto et al., 2020) unquestionably take a part in their complex decision-making process. Moreover, studies find parents have a substantial impact on their children’s education decisions (Tillman, 2015; Workman, 2015; Rowan-Kenyon et al., 2016). However, more than half of Generation Z members (i.e., prospective students) said they consider their family members’ opinions and perspectives, but, when making decisions, they still want to be listened, valued, and involved (Seemiller and Grace, 2016). Furthermore, when potential students have thousands of colleges to choose from, branding is one of the most influential ways to sway their decision. A higher education’s brand is the expression of its defining characteristics that set it apart from the competitor, demonstrate its capacity to satisfy the needs of students, build conviction in its capacity to convey a specified type and quality of higher education, and assist prospective students to be well-informed about enrollment (Sujchaphong et al., 2017).

As a brand, higher education institutions need to explore the way they organize their social media posts and content to connect with prospective students. Brands shouldn’t be afraid to actively communicate with customers and strongly promote their products on social media to raise levels of behavioral engagement (Malhotra and Malhotra, 2012). However, organizations need to be careful because the hard-selling approach simply doesn’t work on social media. Their intention when they open social media is not to see ads milling about in their feeds. Lou et al. (2019) suggest that non-hard-sell branded content plays a crucial part in engaging with customers, boosting customer brand loyalty, and fostering long-term brand building. considering that prospective students may have never had any contact with a higher education, Dessart (2017) suggests that during the early phases of community building, it is even necessary to provide topical content that is unrelated to the business.

In the context of marketing activity content and posts, Kim and Ko (2012) initially developed the concept of social media marketing activity (SMMA), which emphasizes that the relationship between enterprises and their customers on social networks must be enjoyable, interactive, exhibit the latest trend, be customized among different customer segments, and demonstrate word-of-mouth. Other than mentioned by Kim and Ko (2012) and Yadav and Rahman (2018) argue that SMMA in the perspective of e-commerce, informative posts related to the brand are necessary. Hence, the SMMA concept is very likely to be adapted as a benchmark for organizing higher education’s social media and pleasantly reaching prospective students. SMMA is based on the idea that differentiating communication characteristics into functional (cognitive) and emotional (affective) values is an effective technique to express the higher education brand (Palacio et al., 2002).

The general concept of applying social media to engage with potential students through a branding strategy is convincing. However, no prior research has investigated this topic in-depth, specifically on how higher education institutions’ social media should be managed. Meanwhile, consumer engagement is often recognized as an important sign of digital customer relationship management (Hao, 2020), which becomes a trigger for customer buying intentions and decisions (Kumar et al., 2016). Davis et al. (2002) suggest that pre-purchase interactions must be planned to influence consumer perceptions and expectations of the brand, raise brand awareness, and promote its relevance. Organizations should tell potential customers why their brand is better than the competition and how it can help them get what they want and need.

This research extends the existing literature and provides empirical evidence about how social media posts and content influences consumer engagement behavior via brand equity. The current SMMA idea provided by Kim and Ko (2012) will be explored and developed further, with the addition of dimensions that complement it, to reinforce its role even more. This study proposes the SMMA concept as a benchmark or pillar in organizing higher education’s social media to generate positive effects on brand equity. Furthermore, common opinion says that customer engagement comes after brand equity (brand awareness and brand image). Thus, it is important to look into how the brand equity created by SMMA affects how customers behave.

## Literature review

### Social media marketing activities

The initial concept of SMMA was developed by Kim and Ko (2012). They characterize SMMA as a mutual communication that tries to evoke empathy from adolescent users toward established luxury fashion brands. They identify SMMA comprising of five features: entertainment, trendiness, customization, interaction, and word of mouth. According to their findings, SMMA improves customer equity elements (value, relationship, and brand) in the setting luxury apparel business. Many researchers have used this SMMA framework to evaluate cases in a variety of contexts and settings, including brand communities (Chen and Lin, 2019), the leather industry (Khajeh Nobar et al., 2020), Korean cosmetics (Choedon and Lee, 2020), online shopping (Zarei et al., 2021), ride-hailing apps (Moslehpour et al., 2021), Facebook coffee-shop page (Ibrahim et al., 2021), and e-brands (Chen and Qasim, 2021). Most of them consider the SMMA dimensions by Kim and Ko (2012) as a robust SMMA construct. Meanwhile, Yadav and Rahman (2017) formulate the updated dimension and scale of SMMA in the context of e-commerce. They assert that because e-commerce represents a novel industrial setting in terms of both consumer and industry characteristics, a distinct scale to assess perceived SMMA is required. They offer an updated dimension of SMMA: interactivity, personalization, informativeness, trendiness, and word-of-mouth, in which there are some differences from the dimensions proposed by Kim and Ko (2012). Based on the aforementioned study, there are no definite dimensions of SMMA that can explain the general setting and context. Thus, the dimensions of SMMA follow the respective industries.

This study tries to expand the perceived SMMA by developing its dimension that is relevant to the perspective of marketing of higher education. The comprehensive view of SMMA by Kim and Ko (2012) as well as Yadav and Rahman (2018) are further discussed in this study. The dimensions of interactivity, trendiness, and WOM were involved in both studies, as well as customization and personalization dimensions, which are almost similar in definition, were adopted in this study. The dimensions of entertainment mentioned by Kim and Ko (2012) and informativeness mentioned by Yadav and Rahman (2018) were also adopted. Thus, this study used entertainment, information, interactivity, personalization, trendiness, and word-of-mouth (WOM) to describe the SMMA. These dimensions guide how the university-related (de Vries et al., 2017) or non-university-related (Dessart, 2017) posts or content are shared on social media.

Additionally, perceived institutional credibility is relevant to this discussion. Higher education credibility is one of the most discussed and increasingly practiced in the context of new student admissions. The notion of “institutional credibility” refers to the ranking, press reviews, and other information that demonstrates the university’s status and reputation (Lim et al., 2018). According to previous studies, perceived institutional credibility is a significant component in student decision-making while pursuing an education (Nguyen and Leblanc, 2001; Hemsley-Brown and Oplatka, 2006; Kaushal and Ali, 2019). Internal and external stakeholders are increasingly demanding improved outcomes in research, education, knowledge handover, employment, and group engagement (Miotto et al., 2020). Therefore, considering credibility dimensions as part of the perceived SMMA dimension is essential to creating a comprehensive view of SMMA from the higher education perspective. Table 1 exhibits the definition of each SMMA dimension, as well as Table 2 presents the operational definition of SMMA used in this research.

**TABLE 1:** Definition of SMMA dimensions.

Dimensions	Definition	References
Entertainment	The extent to which social media posts and content are entertaining, fun, or enjoyable.	Kim and Ko (2012)
Informativeness	The extent to which social media posts and content show accurate, useful, and comprehensive information.	Yadav and Rahman (2017)
Interactivity	The extent to which social media posts and content demonstrate customers’ sharing ideas, views, or direct interactions with the organization itself or other customers.	Kim and Ko (2012)
Personalization	The extent to which social media postings and content show personalized material to meet a customer’s preferences.	Yadav and Rahman (2017)
Trendiness	The extent to which social media posts and content demonstrate current events, breaking news, or hot debate topics.	Kim and Ko (2012)
Word-of-Mouth	The extent to which social media posts and content demonstrate positive testimonials from students, lecturers, partners, or stakeholders.	Adjusted to the research context
Perceived Institution Credibility	The extent to which social media posts and content demonstrate the organization’s achievement or reputation.	Adjusted to the research context

**TABLE 2:** Operational definition.

Construct	Operational definition
Higher Education’s SMMA	A tool used to manage social media in higher education, such as Instagram, Facebook, Twitter, Youtube, and Blog, to attract potential students in a way that is entertaining, interactive, informative, personal, shows the latest updates or news, emphasizes word-of-mouth, and shows the institution’s credibility.
Higher Education Brand Equity	The value of a higher education institution’s brand comprises a prospective student’s awareness of a higher education institution’s brand and the image that they make with the higher education institution.
Prospective students’ behavioral engagement	The amount of time, effort, and energy a potential student spends reading, interacting with, and feeling connected to posts or content related to or unrelated to higher education, whether they were made by a higher education initiator or by the prospective student themselves. This is because they want to get closer to each other and find out more about college.

### Brand equity

The idea of brand equity is based on two strongly held beliefs established by Aaker and Keller, which have so far led the majority of researchers. Aaker (1992) identifies brand equity as a brand’s assets and liabilities, but does not specify whether they are consumer- or customer-based. He proposes a new terminology for brand equity: consumer-based brand equity (CBBE), which entails of four elements: brand loyalty, brand association, brand awareness, and perceived quality. Referring to Keller (1993), CBBE is defined as the differential effect of brand knowledge on consumer reaction to brand marketing. His idea of brand equity is the most important one, because it connects its two parts, brand awareness and brand image. However, Aaker (1996) mentions that the concepts and dimensions of brand equity may develop or change as a consequence of the distinctive nature of various product contexts. Yet, the researchers still need to provide evidence for the relevance and necessity.

In this study, brand equity follows SMMA as its antecedent and as a construct that affects prospective students’ engagement intentions. Given that deciding on the right college needs a long consideration, engagement refers to a prospective student’s attitudes toward the higher education institution that occur in the primary steps of their decision-making process. Based on Aaker (1996), he implies that brand awareness is the primary factor influencing customers’ perceptions and attitudes. Since the branding strategy comes before attitudes (prospective students’ engagement intention), the dimension of CBBE proposed by Aaker (1992) has no relevance in this study. This is supported by Tasci (2016, 2018), who posits that when he tested the CBBE model’s cross-brand validity, he used brand familiarity (awareness) and brand image as the launchpad elements that highly influence the whole model he tests. Tasci (2021) indicates that the brand equity components (brand loyalty, brand association, brand awareness, and perceived quality) were not equal in one line. He reconstructed the perception of brand equity by placing perceived quality and brand loyalty as components that are influenced by brand awareness and brand image. Grounded on above discussion, the brand awareness and brand image as a components of brand equity were used in this study. Table 2 demonstrate the operational definition of brand equity used in this research.

Creating brand equity has obvious advantages. Brand equity is the perceived value of a brand in the minds of consumers. “Brand equity” is a signaling phenomenon that ensures the product’s high quality (Hazée et al., 2017). According to Carvalho et al. (2020) brand equity has become an essential concept for higher education institutions. Choosing a higher education is a long-term personal investment for prospective students that will decide their professional future. As a result, brand equity can act as a risk mitigator, influencing the choice of a higher education institution (Mourad et al., 2011). According to Royo-Vela and Hünermund (2016), when information reaches a prospective student, it is more likely to be favorably received and responded to because of prior positive associations with the brand. As an outcome, prospective students are likely to compare institutions’ brand equity while selecting a higher education institution.

#### Brand awareness

Rossiter and Percy (1987) were the first to define Brand awareness is defined as customers’ capacity to identify the brand in a variety of situations. This shows how strong the brand node, or memory of this brand is. High levels of consumer awareness are expected to facilitate information processing, decrease risk and ambiguity, and foster favorable feelings toward an object (Tasci and Boylu, 2010). Keller (1993) states that CBBE arises once the customer is aware of the brand, is familiar with it, and has positive, strong, and distinctive memories associated with it. Recently, Bergkvist and Taylor (2022) Using previous research and current advertising industry trends, the new explanation of brand awareness is the possibility that a individual would remember a brand name, a product classification, or a category requirement in a range of brand-relevant situations. Brand awareness is a way to measure how well customers can tell the difference between a company’s name, products, or services and those of its competitors.

#### Brand image

A brand image makes an impression. According to Keller (1993), brand image is described as a brand’s opinions. The brand’s image acts as the focus point for information in customers’ memories about the brand’s advantages, benefits, and sentiments (Aaker, 1992, 1996). The brand image is what customers think and feel when they hear or see an institution’s or brand’s name (Mothersbaugh et al., 2020). Rethinking the idea of brand image is necessary given the rapid technological improvements, digital (online) innovations, and societal and environmental constraints (Gürhan-Canli et al., 2016). A brand image may also be looked at as a set of customer ideas about a product or service that are typically arranged to convey a meaning.

### Behavioral engagement

Engagement can also be thought of as the interaction with content. In this research, behavioral engagement is more than just customers’ intention to follow a social media account. The total of time, attempt, and energy spent by a customer on a brand during a given consumer-brand encounter is referred to as “digital behavioral engagement” (see Table 2). Vivek et al. (2014) demonstrate that this kind of relationship is the extent to which an individual engages with an organization’s services or activities and feels connected to them, which is begun by the customer or the organization. Customer engagement is valuable to firms because it generates positive outcomes, including self-brand relationship and brand usage intention (Harrigan et al., 2018). Engaging content is a criterion for the beneficial effects of social media success (i.e., the number of reaches, likes, and shares) that may turn into positive sales performance (Ha et al., 2016) and branding (Hudson et al., 2016).

In the context of higher education, when a prospective student engages with the higher education institution’s social media, the higher education has an opportunity to convince them by sharing beneficial information. Because customers spend so much time on social media, there’s a significant likelihood they’ll participate in social brand activities (Osei-Frimpong and McLean, 2018). The engaging prospective student will enjoy and show a positive attitude toward posts issued because of the desire to learn more about this higher education institution. In other words, the possibility of prospective students choosing this higher education has increased.

## Hypotheses development

SMMA, with its dimension, has been addressed as a predictor of customer-based brand equity in many settings (Kim and Ko, 2012; Godey et al., 2016; Yadav and Rahman, 2017; Ibrahim et al., 2020). Furthermore, in social media marketing research, brand equity has been analyzed as a second-order object with two dimensions (Barreda et al., 2015; Seo and Park, 2018), namely brand image and brand awareness. Ibrahim et al. (2020) recently reported a meta-analysis study looking at the correlation between SMMA and brand equity, as well as purchase intention. According to the findings, there is a substantial relationship between SMMA and brand equity. The above discussion supports the premise that, when a student is deciding which institution to attend (i.e., tied to a higher education brand), prospective students will heavily rely on social media to assist and guide them in their college selections. As a result, having a robust social strategy is essential since, in this study, SMMA was set up to be a tool for social media management. Thus, this study proposed H1.

**H1:** SMMA has a significant influence on brand equity.

Schreiner et al. (2021) uncovered that the attractiveness of a social media post and its high media richness (e.g., the inclusion of elements like photos or videos) positively influenced engagement behaviors. While enjoyable content induced more sharing activity than incentive content, incentive content elicited more “like” behavior (Luarn et al., 2015). Customers in the tourist industry believe that postings or content linked to their interests are more pleasing, fascinating, or amusing, prompting them to share, like, or comment on it (Onofrei et al., 2022). In the luxury brand context, customers’ perception of the relevance of the post or content is one of the key drivers of their engagement on Facebook and Instagram (Bazi et al., 2020). A humorous type of post or content that isn’t specifically about a product is recognized as being crucial for increasing customer engagement (Ge and Gretzel, 2017). This research proposes SMMA with its dimensions as a strategy for organizing higher education’s social media. Using SMMA, the content or post on social media has to be entertaining, share relevant information, be interactive, personal, demonstrate recent updates or news, emphasize word-of-mouth, and show the institution’s credibility. Therefore, with such diverse and not boring content, it can be expected that prospective students will incline to be more involved, as one of their initial efforts to find out more about the college they want to go to. Thus, H2 is proposed.

**H2:** SMMA has a significant influence on engagement behavior.

Maintaining consumers’ positive associations with brands has become a crucial component of a marketing strategy to encourage behavioral engagement. A recent study discovered that CBBE influences customers’ engagement behavior in favor of brands on social media (Schivinski et al., 2019). In the short and medium terms, a brand with engaged customers will experience beneficial financial and non-financial effects (van Doorn et al., 2010). In the setting of a luxury brand, both brand equity dimensions (brand image and brand awareness) have been shown to be significant predictors of customer engagement behavior (Gallart-Camahort et al., 2021). Moreover, the way the brand communicates with the customers confidently impacts the degree of customer brand engagement (Gómez et al., 2019). A well-thought brand, along with a set of effective techniques, will help higher education to easily interact with users on social networks. Brand equity comes first when designing a brand strategy. The prospective student should be able to recognize higher education names among competitors and recall them. Higher education should work to increase brand engagement in order to pique the interest and decision-making of potential students. Thus, H3 is proposed.

**H3:** Brand equity has a significant influence on engagement behavior.

## Research methodology

### Data collection and sample profile

From February 2022 to June 2022, data was collected via a web-based online questionnaire. The close-ended statement was used to ask for the level of the respondent’s agreeableness in the form of a 1 (strongly disagree) to 7 (strongly agree) point Likert scale. Purposive sampling was utilized to explain a certain subject, idea, or phenomenon based on the needs of the individual informants (high school students). However, the study’s population size was unknown. Thus, in business research, a convenience technique was used since it provides an accessible form of data gathering.

Afterward, a link to the measurement items was distributed to Indonesian third-grade high school students. The research objective is to assess what makes a prospective student decide to build engagement with their future college on social media in order to strengthen their decision about which college they want to go to. In this research context, the prospective students refer to high school students. Thus, third-grade high school students were considered an appropriate sample for this study.

The study’s objectives, researchers’ contacts, data collection methods, data protection concerns, and ethical considerations were all covered on the questionnaire’s front page. In all, 371 answers were received. However, 356 valid responses were accepted for further analysis after 15 questionnaires had incomplete responses. The gender distribution of respondents is quite even. Meanwhile, for the age distribution of respondents, in Indonesia, the average age of 3rd grade high school students is 17–19 years old, but most of them are 17 years old. Therefore, in this study, respondents aged 17 years dominated. Table 3 displays the demographic information for the research’s sample.

**TABLE 3:** Demographic information.

Dimension	Item	Frequency	Percentage (%)
Gender	Male	166	46.63
	Female	190	53.37
	Total	356	100
Age	17 years old	297	83.43
	18 years old	43	12.08
	19 years old	16	4.49
	Total	356	100

### Measurement item

The measurement items of the previous study were employed as the basis for this research’s measurement items due to the validity of the questionnaire contents have been properly verified yet adjusted for this study’s context and settings. The measurement items of SMMA dimensions were taken from the statement that was used in Kim and Ko (2012) and Yadav and Rahman’s (2017) research. However, because the word-of-mouth notion in the two above mentioned studies was slightly different, the author devised the measuring item grounded on the concept given in the previous section. The institution credibility items as a complement to the SMMA concept in the context of higher education were retrieved from Kethüda (2022) and Merchant et al. (2015). Brand equity measurement items, which included its two elements (brand awareness and brand image), were extracted from Seo and Park (2018). Finally, the measurement items of behavioral engagement were adapted from various sources (Mirbagheri and Najmi, 2019; Ni et al., 2020; Cheung et al., 2021) and being adjusted to the context of this research.

A pilot study was conducted to evaluate the responsiveness’s accuracy and consistency. A pretest was conducted involving 20 people from the target group (high school students). Furthermore, the questionnaire was evaluated from a marketing professor’s perspective. Moreover, language experts who understand both languages (English and Bahasa Indonesia) were involved. Given that the questionnaire reference was in English, while the question or statement was intended for Indonesians, it must be interpreted clearly. Some changes were made to reduce the ambiguity (Table 4).

**TABLE 4:** Final measurement item.

Construct	Questions/Statements
**SMMA dimensions**	
**Entertainment (Kim and Ko, 2012)**	
ENT1	I prefer HE’s social media to post fun things.
ENT2	Entertaining content on HE’s social media seems interesting to me.
**Informativeness (Yadav and Rahman, 2017)**	
INF1	I enjoy reading insightful posts on HE’s social media.
INF2	Useful information on HE’s social media seems interesting to me.
INF3	The information provided by HE’s social media is comprehensive
**Interactivity (Kim and Ko, 2012; Yadav and Rahman, 2017)**	
INT1	I’m impressed when HE uses social media to regularly interact with its followers.
INT2	I like interactive activities when looking at a HE’s social media posts or content.
INT3	It’s interesting if HE’s social media posts encourage followers’ conversation.
**Personalization (Yadav and Rahman, 2017)**	
PER1	I’m pleased if HE’s social media posts align with what I want to see.
PER2	I feel connected when HE’s social media posts as per my preferences.
PER3	I prefer it if the information shared by HE on social media is related to my interests.
**Trendiness (Kim and Ko, 2012; Yadav and Rahman, 2017)**	
TRE1	I’m interested if HE’s social media shares the latest information.
TRE2	I think recent info, news, or events are important for HE’s social media to post.
TRE3	I prefer it if HE’s social media shares trendy information.
**Word-of-mouth (developed by author)**	
WOM1	A content about alumni success stories really inspires me.
WOM2	Positive comments, impression or testimonials from active students, lecturers, college partners need to be posted in HE’s social media.
WOM3	Reposting a followers-generated-content (i.e., mentions) can broaden my understanding of the higher education.
**Institution credibility (Merchant et al., 2015; Kethüda, 2022)**	
INC1	It is important to post higher education achievements on social media.
INC2	I’m interested in knowing about good higher education activities.
INC3	When I think about a certain higher education, I am reminded of a graduate who has the proper knowledge and skills.
**Brand equity**	
**Brand awareness (Seo and Park, 2018)**	
BRA1	Because of social media activities, I’m always aware of HE’s name.
BRA2	Because of social media activities, I’m aware of the characteristics of a HE.
BRA3	Because of social media activities, I can always remember
	the logo of a HE.
**Brand image (Seo and Park, 2018)**	
BRI1	Through their social media, HE can show a positive image.
BRI2	I can be impressed with HE’s good social media activity.
BRI3	I respect to a HE considering what they post on social media.
**Behavioral engagement (Mirbagheri and Najmi, 2019; Ni et al., 2020; Cheung et al., 2021)**	
BVE1	Following HE’s social media posts can improve my knowledge of HE.
BVE2	I prefer to check out a college’s social media profiles when I want to learn more about it.
BVE3	I would like to read content posted by HE’s on their social media.
BVE4	I’m satisfied with my relationship with a particular HE’s social media account.

## Data analysis

**FIGURE 1:**
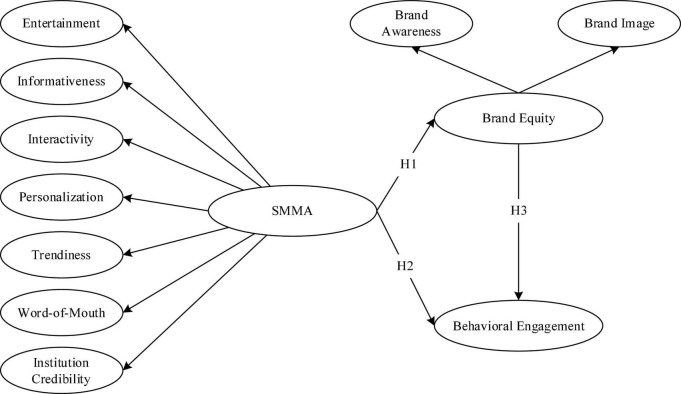
Research framework.

The research framework was developed based on higher-order constructs (Sarstedt et al., 2019). SMMA and brand equity act as higher-order constructs, whereas the respective dimensions act as lower-order constructs. The interaction between the higher-order component and its lower-order components is referred to as the reflective-reflective higher-order type (Ringle et al., 2012) (see Figure 1). In two stages, estimation and partial-least-square structural equation modeling (PLS-SEM) studies were performed. The first step is outer model assessment to assess the reliability and validity of an established model. The inner model assessment comes next to evaluate the hypotheses’ connection. PLS was chosen because it can handle model constructs and measurement items concurrently and is appropriate for examining the causal linkages between construct variables (Petter et al., 2007).

### Outer model and scale validation

An outer model evaluation was conducted to evaluate the quality of the construct. The construct’s quality assessment starts with factor loading, followed by construct validity and construct reliability (see Table 5). The amount to which the item in the correlation matrix correlates with the given main component is referred to as factor loading. Suggested by Hair et al. (2016), the factor loading should be more than 0.50. Hence, no item in the study had a factor loading fewer than the suggested value, hence, the factor loading is not the issue in this research. A reliability analysis was presented to measure the instrument stability and consistence. In this research reliability test, Cronbach’s alpha and composite reliability were employed. Both Cronbach’s alpha and composite reliability ranged from 0.752 to 0.903 and 0.871 to 0.966, respectively, which is greater than the suggested limit of 0.70 (Hair et al., 2014). Therefore, these research constructs are reliable.

**TABLE 5:** Factor loading, construct validity, and reliability.

Item	Factor loading	Cronbach’s alpha	Composite reliability	AVE
ENT1	0.881	0.752	0.889	0.801
ENT2	0.908			
INF1	0.915	0.903	0.939	0.838
INF2	0.938			
INF3	0.892			
INT1	0.805	0.821	0.894	0.737
INT2	0.885			
INT3	0.884			
PER1	0.888	0.834	0.900	0.751
PER2	0.905			
PER3	0.804			
TRE1	0.939	0.948	0.966	0.905
TRE2	0.953			
TRE3	0.962			
WOM1	0.869	0.891	0.932	0.821
WOM2	0.926			
WOM3	0.923			
INC1	0.872	0.876	0.923	0.800
INC2	0.910			
INC3	0.901			
BRA1	0.816	0.804	0.884	0.718
BRA2	0.872			
BRA3	0.854			
BRI1	0.833	0.778	0.871	0.693
BRI2	0.867			
BRI3	0.794			
BVE1	0.694	0.822	0.883	0.654
BVE2	0.817			
BVE3	0.837			
BVE4	0.877			

ENT, entertainment; INF, informativeness; INT, interactivity; PER, personalization; TRE, trendiness; WOM, word-of-mouth; INC, institution credibility; BRA, brand awareness; BRI, brand image; BVE, behavioral engagement.

The validity of constructs was tested using two approaches: convergent validity and discriminant validity. Convergent validity is a parameter that ensures that two or more measures of the same entity differ significantly (Bagozzi et al., 1991). A valid construct (item converge to measure the underlying construct) is represented by an AVE value of more than 0.50 (Fornell and Larcker, 2018). In this research, the AVE value grater was 0.50, so convergent validity is established. Discriminant validity testing was done to make sure that the measurement items were different and distinct. Each measurement item needs to be unique and not highly correlated with each other (Bagozzi et al., 1991). Agreeing to Fornell and Larcker (2018), discriminant validity is demonstrated when the square root of AVE for each concept is larger than its correlation with all other constructs. In this research, the construct’s square root AVE (in bold) was shown to be stronger than its correlation with the other constructs. As a result, the test offers substantial support for establishing discriminant validity (see Table 6).

**TABLE 6:** Forner and Larcker discriminant validity.

	BRA	BRI	BVE	ENT	INC	INF	INT	PER	TRE	WOM
BRA	**0.848**									
BRI	0.643	**0.832**								
BVE	0.723	0.669	**0.809**							
ENT	0.591	0.613	0.606	**0.895**						
INC	0.512	0.491	0.466	0.490	**0.895**					
INF	0.541	0.640	0.550	0.708	0.481	**0.915**				
INT	0.577	0.636	0.626	0.694	0.449	0.780	**0.859**			
PER	0.482	0.577	0.592	0.588	0.462	0.649	0.654	**0.867**		
TRE	0.511	0.579	0.564	0.586	0.451	0.644	0.646	0.714	**0.951**	
WOM	0.592	0.544	0.612	0.556	0.505	0.504	0.558	0.532	0.492	**0.906**

ENT, entertainment; INF, informativeness; INT, interactivity; PER, personalization; TRE, trendiness; WOM, word-of-mouth; INC, institution credibility; BRA, brand awareness; BRI, brand image; BVE, behavioral engagement. The bold character is AVE square root.

As part of the measurement model evaluation, these higher-order constructs (SMMA and Brand Equity) are also evaluated. The reliability and convergent validity of each of these constructs were evaluated. Furthermore, as advised by Sarstedt et al. (2019), the higher order construct was examined for discriminant validity with the other lower order construct in the research. The outcomes of the higher order construct’s reliability and validity demonstrate that both reliability and validity were established. The result of the higher order reliability (Cronbach’s alpha and composite reliability) is larger than 0.70 and the AVE is larger than 0.5 (see Table 7). Moreover, the result of Fornell and Larcker’s discriminant validity indicates that AVE’s square root (in bold) of the construct is higher than its correlation with all other constructs (see Table 7).

**TABLE 7:** Higher-order construct reliability and convergence validity, as well as the discriminant validity of Fornell and Larcker criterion.

	Cronbach’s alpha	Composite reliability	AVE	BE	SMMA
BE	0.783	0.902	0.821	**0.906**	
SMMA	0.906	0.926	0.642	0.778	**0.801**

BE, brand equity; SMMA, social media marketing activities. The bold character is AVE square root.

Additionally, CMV (common method variation) might be a significant issue for any self-reported data on SEM. The presence of CMV in the dataset indicates that the results are not analytically valid. To address this problem, this study used Harman’s one-factor examination to determine the presence of the CMV (Podsakoff et al., 2003). A single factor is extracting 45.882% of the total variance. Since it is fewer than 50%, it can be concluded that there is no threat of CMV.

Lastly, to understand the overall quality of the suggested model, this study estimated the Goodness of Fit (GOF) as Tenenhaus et al. (2005) suggested. The GOF is determined as follows:


(1) $$GOF=\sqrt{\bar{AVE}\times \bar{R^{2}}}=\sqrt{0.772\times 0.609}=0.686$$

This research GOF value is 0.686, which surpasses the cut-off threshold of 0.36 for a substantial outcome size (Wetzels et al., 2009).

### Inner model result

**FIGURE 2:**
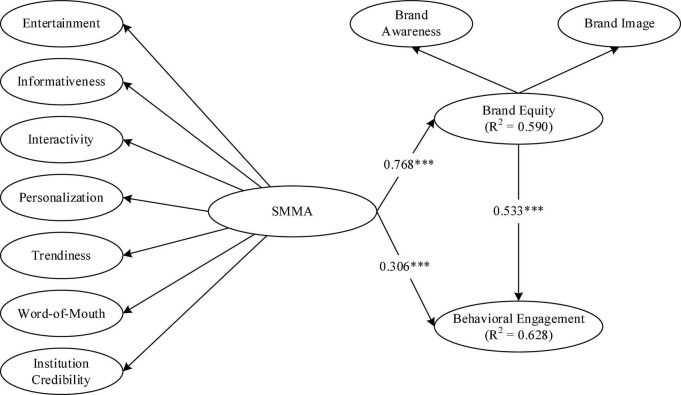
Standardized path coefficients and significance of inner model. ^***^p < 0.001.

Structural equation modeling, or inner model, is an assessment of the hypothesized relationship to substantiate the proposed hypothesis. The weight of each path coefficient was estimated using bootstrapping. Re-sampling data was utilized to estimate the values, and the calculated values were more exact than the usually used limit approximation value (Purvis et al., 2001). As a result, this technique was utilized in this study to assess the significant relationships between variables (Table 8). Figure 2 present the tested hypotheses in this study. The analysis shows that SMMA had a significant effect on brand equity (β = 0.768; *t*-value = 22,976; *p* < 0.001). Thus, hypothesis 1 was supported. Furthermore, SMMA had a significant impact on behavioral engagement (β = 0.306; *t*-value = 5,098; *p* < 0.001), supporting hypothesis 2. Finally, brand equity had a significant consequence on behavioral engagement (β = 0.533; *t*-value = 0.533; *p* < 0.001).

**TABLE 8:** Summary of hypothesis testing result.

Hypothesis	Path	Standardized path coefficient	*t*-value	Conclusion
H1	SMMA → BE	0.768***	22.976	Supported
H2	SMMA → BE	0.306***	5.098	Supported
H3	BE → BE	0.533***	9.016	Supported

BE, Brand Equity; SMMA, Social Media Marketing Activities; BE, Behavioral Engagement. ***p < 0.001. Number of bootstrap samples = 10,000.

The inner model assessment is also used for estimating the R-square and path coefficient. Figure 2 presents the R-square value and path coefficient of this research’s structural model. R-square statistics reflects the variation in the dependent variable is clarified by the independent variable. Simply expressed, it refers to how much conversion in the dependent variable can be explained by one or more independent variables. In this research, brand equity was influenced by SMMA with an R-square value of 0.590. Meanwhile, the behavioral engagement variable was influenced by SMMA and brand equity with an R-square value of 0.628. This result explains that a 59.0% change in brand equity was caused by SMMA, while a 62.8% change in the behavioral engagement variable was caused by SMMA and brand equity. Since Hair et al. (2013) recommend that the R-square values of 0.75, 0.50, and 0.25 for the dependent latent variable can be described as substantial, moderate, and weak, respectively. Thus, the outcome indicates that both dependent variables (brand equity and behavioral engagement) are moderately explained by each independent variable. The path coefficients (or β-values) represent the hypothesized relationships or the strength of the relationship between independent and dependent variables. The path coefficient close to +1 indicates a strong positive relationship. The closer the path coefficient’s value to 0 means a weaker relationship. The two paths (SMMA → BE and BE → BVE) show strong relationships (0.768 and 0.533, respectively), whereas the other path (SMMA → BVE) shows a weak relationship (0.306).

### Mediation test

The mediation analysis was attempted to explore the mediation consequence of brand equity in the connection between SMMA and behavioral engagement in order to determine whether the mediation model provided in this research was statistically considerable. The results (Table 9) show that SMMA had a significant total effect on behavioral engagement (β = 0.715; *t*-value = 21.858; *p* < 0.001). With the mediating variable (brand equity) included, the impact of SMMA on behavioral engagement remains significant (β = 0.306; *t*-value = 5.098; *p* < 0.001). Through brand equity, the indirect effect of SMMA on behavioral engagement was found to be significant (β = 0.409; *t*-value = 8.284; *p* < 0.001). This calculation reveals that the relationship between SMMA and behavioral engagement is partially mediated by brand equity.

**TABLE 9:** Mediation test result.

Total effect of SMMA → BVE		Direct effect of SMMA → BVE		Indirect effect of SMMA → BVE		
β	*t*-value	β	*t*-value		β	*t*-value
0.715***	21.858	0.306***	5.098	SMMA → BE →BVE	0.409***	8.284

BE, brand equity; SMMA, social media marketing activities; BE, behavioral engagement. ***p < 0.001. Number of bootstrap samples = 10,000.

## Discussion

The interest in studying the benefits of social media on an organization was increasing, as well as how they played a role in the organization’s brand building (Zollo et al., 2020). Nonetheless, researchers need to work harder to discover real-world instances of how social media impacts brand equity and how this shapes other valuable branding goals, such as getting customers to act in a certain way. Agreeing to Dwivedi et al. (2021), social media is always in beta mode due to its constant change and consistency in releasing new functions and features. This makes analyzing social media as a research topic always challenging. Moreover, Schultz and Peltier (2013) added that due to the various conceptual and measurement issues, research must be expanded in the realm of social media. This research fills an essential divergence in the literature by conducting a research on how higher education uses social media.

This study delivers suggestion of how important higher education’s social media activities are in shaping their brand awareness as well as brand image, which results in prospective students’ behavioral engagement. The result shows that the SMMA with its dimensions has a clear impact on higher education brand equity, which consists of brand awareness and brand equity. The adoption of various social media channels is essential for developing an effective higher education brand since the target is a “digital native.” Moreover, the results demonstrated that brand equity has a significant influence on prospective students’ behavioral engagement. While customers pay attention in a positive way, there is a willingness to learn more about a particular product. In the prospective student context, that kind of desirability plays a part in their long-decision-making process while choosing a college that they want to attend.

### Theoretical implication

This research helps us learn more about the marketing of higher education in a multiple of approaches. First, this research adds to the body of knowledge on social media marketing by creating an instrument to measure how SMMA is regarded in the perspective of higher education. The current SMMA concept remains adopted, but there are some adjustments in the concept of word-of-mouth and attaching the perceived institution credibility. Previously, word-of-mouth as a part of SMMA dimensions (Kim and Ko, 2012; Yadav and Rahman, 2017; Seo and Park, 2018) was defined as the creation of shareable content to generate customers’ positive word-of-mouth (user-generated-content). However, in the context of higher education, this understanding still needs to be expanded. In addition to the understanding described above, the word-of-mouth concept was developed into the degree to which prospective students perceive positive word-of-mouth from higher education stakeholders who recommend and share experiences about the institution on social media. Additionally, institution credibility needs to be employed as the complement of perceived SMMA in the higher education context, since institution credibility is considered as an notable aspect in prospective students’ decision-making path when selecting a college to attend (Nguyen and Leblanc, 2001; Hemsley-Brown and Oplatka, 2006; Kaushal and Ali, 2019).

Second, this examination adds to the existing research by showing a complete framework for how SMMA affects brand equity and how customers behave when they interact with an organization. Although past literature acknowledged the importance of certain SMMA aspects (Godey et al., 2016), our empirical research demonstrates their relative relevance and demonstrates that when designing social media operations, all five factors should be evaluated as a whole. All five dimensions of SMMA stand out from the perspective of a potential student.

Third, this study analyzes the evolution of the notion of behavioral engagement in the perspective of higher education. A previous study defined engagement intention or participation intention as a customer’s propensity to follow, like, remark on, or share an organization’s generated content (Onofrei et al., 2022). However, in the perspective of prospective students for higher education, behavioral engagement denotes to their propensity to interact with an organization’s services or activities and feel connected, which is initiated by either the consumer or the organization. This eagerness stems from a desire to learn more about their future educational institution.

Fourth, the mediation test result shows that brand equity could be a strong factor in the link between SMMA and behavioral engagement. The direct effect path coefficient of SMMA and behavioral engagement is weak. While the path coefficient of its total effect is strong. Supported by significant indirect effect of SMMA to behavioral engagement, this result indicates that the relationship between SMMA and behavioral engagement is strong, but this strong relationship is transmitted through a mediator, which is brand equity. Thus, brand image and brand awareness, as dimensions of brand equity, play an essential mediating role in SMMA and behavioral engagement.

Finally, this is the first research to examine the influence of social media marketing on brand equity and, as a result, essential consumer behavioral engagement in the perspective of higher education. By using the novel data type, this study contributes to the area of research in a variety of ways.

### Managerial implication

The research findings shed light on how higher education social media managers should manage their social media to generate the most beneficial impact for the institution, which is getting more new students. Higher education suggested investing more resources in managing social media since this is the most effective tool for communicating its competitive advantage. Moreover, since the audience is Generation Z, reaching them through social media is promising.

Based on the test result, all the dimensions of SMMA need to be involved to create a big picture of higher education’s social media activities. It gives higher education social media managers ideas and direction (as a pillar) on how to manage, organize, and arrange their social media posts and content. Since it has become more effective in creating customer engagement (Lou et al., 2019), the soft-selling approach has become the foundation for implementing the SMMA dimension in creating posts and content, even having to post stuff that has nothing to do with the brand (Dessart, 2017). But, in order to foster the customer’s knowledge, a hard-selling approach is needed at some points (Northcott et al., 2021). All this effort is meant to create valuable content and posts. Not only blasting out consumers with things that the organization wants, but the organization must also be able to accommodate what customers want to see. Furthermore, the SMMA dimension as the basis for the idea of creating content and posts does not have to rigidly refer to only one dimension. It may be that a single post or piece of content contains more than one characteristic or dimension. For example, a post or content may be entertaining, informative, and interactive. Aside from that, the SMMA dimension could be applied to a variety of posts and content, including photos, tweets, videos, infographics, blogs, articles, and so on, and could be shared across social media platforms.

Hypothesis 1 demonstrates that these sets of SMMA positively influence brand equity. Prospective students increasingly depend on social media to help them make college decisions. As a result, having a solid social media strategy is critical. By applying the SMMA concept, it is able to draw out what distinguishes a university from others and give prospective students a better idea of their brand and its offerings. Higher education can enhance brand awareness on social media at a comparatively low cost. However, higher education awareness will not increase overnight. But, with persistence, consistency, and the use of the aforementioned SMMA strategies, higher education will be well on its way to establishing a brand that is instantly recognizable by potential students. Moreover, prospective students create an impression of higher education based on the current perception of higher education in the market. By using the SMMA strategy, social media can create a brand image for higher education and build trust among potential students.

Hypothesis 2 gives evidence that the SMMA has a positive influence on prospective student behavioral engagement. Which means, prospective students will be looking to a school’s social media pages for important updates and to get a better impression of what life on their campus is like. This research suggests the higher education social media manager should create engaging content and posts. The concept of SMMA gives guidance on how to create engaging content.

Hypothesis 3 states that brand equity, with its dimensions (brand awareness and brand image), has a significant impact on prospective student behavioral engagement. Prospective students are eager to learn more about higher education when they recognize it and perceive it to have a positive image. Moreover, SMMA influences prospective students’ behavioral engagement through brand equity. According to this finding, higher education may use social media to increase interactions with prospective students, but they need to rely on more established techniques (such as institution image and environment) to persuade them to engage with them. Lastly, this study’s mediation test was statistically relevant. The theoretical analysis based on the results shows that brand equity plays an important mediating role in SMMA and behavioral engagement. These results show the higher education social media manager that when arranging or creating social media content, they need to think about what topics will not only get prospective students’ attention but also improve the university’s image. Social media managers should not be careless about what they post on social media, especially in higher education’s social media account context. For example, although this research allows posting funny entertainment content, it must also pay attention to aspects of authority, formality, and norms that are very closely related to a university, and not post any unpolite memes that will actually degrade the image of the higher education.

## Research limitations and future study

As Gen Z gets old enough to go to college, it has never been more important for colleges and universities to have a presence on social media. This research looks at the expanding role of social media in higher education and explores how higher education can use social tools to build their institutional reputation and foster a sense of engagement among their prospective students. Based on the results, prospective students increasingly depend on social media to help them make college decisions. Yet, just blasting out recruiting materials on higher education social feeds every day or doing the same type of content every week is not working. By adding the SMMA dimension, they can help higher education improve their brand awareness and image among potential students. As a result, by recognizing higher education and perceiving a good institution image, prospective students are willing to engage with it and make their behavioral engagement as an element of their decision-making process in choosing a college. Despite efforts to create a complete conceptual model and evaluate fresh data, a number of defects may be found in future research. First, comparing the results of prospective students’ and current students’ is interesting since both are the audience of higher education social media. The different purposes of their intention to engage may vary, driving different results as well. Second, future research needs to consider more customer behavioral aspects as a result of SMMA, specifically in the higher education circumstances. Given the importance of word-of-mouth in the process of new student recruiting, it is very interesting to figure out how to shape prospective students’ (or current students’) behaviors at the highest level of engagement so that they can voluntarily, actively, and interactively help develop the college brand. Third, we conducted a qualitative study to discover why entertaining, informative, interactive, personalized, trendiness, word-of-mouth oriented, and perceived institutional credibility-oriented posts or content are needed and beneficial from a customer’s perspective, specifically. Fourth, as measures of brand equity, this study used brand awareness and brand image. Upcoming research should integrate other factors such as brand quality and brand associations, as well as reconsider the process of establishing brand equity. Finally, this study’s generalizability extends beyond the higher education sector. While the findings are likely to be valuable in higher education, they may not be immediately transferrable to other businesses. The results’ generality must be validated in different circumstances.

## Data availability statement

The raw data supporting the conclusions of this article will be made available by the authors, without undue reservation.

## Author contributions

AR, OS, AW, and S-CC: conceptualization and writing—original draft, review, and editing. AW: formal analysis, investigation, and visualization. All authors have read and agreed to the published version of the manuscript.
